# Effective Removal of Methylene Blue on EuVO_4_/g-C_3_N_4_ Mesoporous Nanosheets via Coupling Adsorption and Photocatalysis

**DOI:** 10.3390/ijms231710003

**Published:** 2022-09-02

**Authors:** Xia Ran, Li Wang, Bo Xiao, Li Lei, Jinming Zhu, Zuoji Liu, Xiaolan Xi, Guangwei Feng, Rong Li, Jian Feng

**Affiliations:** Engineering Research Center for Molecular Medicine, School of Basic Medical Sciences, Guizhou Medical University, Guiyang 550025, China

**Keywords:** adsorption, photocatalysis, EuVO_4_/g-C_3_N_4_ heterojunction, methylene blue, Langmuir isotherm

## Abstract

In this study, we first manufactured ultrathin g-C_3_N_4_ (CN) nanosheets by thermal etching and ultrasonic techniques. Then, EuVO_4_ (EV) nanoparticles were loaded onto CN nanosheets to form EuVO_4_/g-C_3_N_4_ heterojunctions (EVCs). The ultrathin and porous structure of the EVCs increased the specific surface area and reaction active sites. The formation of the heterostructure extended visible light absorption and accelerated the separation of charge carriers. These two factors were advantageous to promote the synergistic effect of adsorption and photocatalysis, and ultimately enhanced the adsorption capability and photocatalytic removal efficiency of methylene blue (MB). EVC-2 (2 wt% of EV) exhibited the highest adsorption and photocatalytic performance. Almost 100% of MB was eliminated via the adsorption–photocatalysis synergistic process over EVC-2. The MB adsorption capability of EVC-2 was 6.2 times that of CN, and the zero-orderreaction rate constant was 5 times that of CN. The MB adsorption on EVC-2 followed the pseudo second-order kinetics model and the adsorption isotherm data complied with the Langmuir isotherm model. The photocatalytic degradation data of MB on EVC-2 obeyed the zero-order kinetics equation in 0–10 min and abided by the first-order kinetics equation for10–30 min. This study provided a promising EVC heterojunctions with superior synergetic effect of adsorption and photocatalysis for the potential application in wastewater treatment.

## 1. Introduction

With the rapid development of modern industry and the consumption of fossil energy, environmental pollution has become increasingly serious. It is regarded as one of the major hindrances for the sustainable development of human society [1]. The development of renewable and green water purification technology for effective removal of organic contaminants is an extraordinarily problematic undertaking. In recent decades, the strategy coupling adsorption and photocatalytic degradation based on semiconductor catalyst has attracted considerable attention in wastewater treatment due to its high efficiency, low energy consumption, the wide availability of adsorbates, superior recoverability, and less secondary pollution [2,3]. In this strategy, the adsorption process can concentrate organic contaminants from the aqueous solution on the surface of a catalyst. Thereafter, the photocatalytic degradation process can ultimately mineralize organic contaminants to small molecule compounds such as H_2_O and CO_2_. Therefore, the active sites on the adsorbent surface are recovered and utilized for the next adsorption and photocatalysis process. The integration of adsorption and photocatalytic degradation is consequently a renewable and green water purification technology for effective removal of organic contaminants.

Graphitic carbon nitride (g-C_3_N_4_, CN) has been widely investigated in environmental remediation as a semiconductor with impressive merits, including a proper band gap for visible light harvesting, low toxicity, high stability, low cost, and so on [4]. Researchers have reported the adsorption process on CN for wastewater treatment [5,6,7]. However, both the adsorption and photocatalytic degradation performance of CN still need to be improved. Generally, the porous structure and large surface area are beneficial for ameliorating the adsorption capability [3]. The ultrathin and layered structure of CN nanosheets has a higher specific surface area and more abundant adsorption sites than bulk g-C_3_N_4_. Therefore, the exfoliation of bulk g-C_3_N_4_ to obtain ultrathin CN nanosheets is considered to be an effective morphology control technique to promote the adsorption capability [6]. For photocatalysis, on the other hand, the diffusion distance of photoinduced charge carriers is shortened in ultrathin CN nanosheets, which can reduce the recombination of photoinduced charge carriers and boost the photocatalytic performance [8]. In addition, semiconductors with suitable band gaps could be employed to design a g-C_3_N_4_-based heterojunction [9,10,11,12]. The spatial separation of photoinduced charge carriers at the interface in heterojunction can efficiently suppress its recombination. It is expected to further improve the photocatalytic performance of g-C_3_N_4_.

Europium vanadate (EuVO_4_, EV) has been studied as a photocatalyst [13,14], laser material [15], and red phosphor [13,16] for its magnetism, thermal stability, and proper energy band gap. Lin et al. prepared EV with different morphologies by the high-temperature electrochemistry method. This prepared adsorbent exhibited good selective adsorption properties and high removal efficiency for U(VI) [17]. Vosoughifar synthesized EV nanoparticles via a precipitation approach; 82% of methyl orange was eliminated in 80 min under ultraviolet light irradiation [18]. In other research, EV nanoparticles were manufactured via a sonochemical method; 64% of methyl orange was removed after 70 min of UV irradiation [14]. Furthermore, a EuVO_4_-based heterojunction has been synthesized to promote its photodegradation efficiency. He et al. synthesized a V_2_O_5_/EuVO_4_ heterojunction from the aqueous solutions of Eu(NO_3_)_3_ and NH_4_VO_3_, which demonstrated high activity for the photodegradation of acetone under both UV and visible light [19]; 99.4% of acetone could be degraded under visible light. It manifested that a small amount of V_2_O_5_ loaded in EV could significantly enhance the photocatalytic efficiency of vanadates [19,20]. Fe_2_O_3_/EuVO_4_/g-C_3_N_4_ ternary nanocomposites were designed and used to degrade rhodamine B [21]; 80.06% of rhodamine B was removed using visible source, exhibiting higher photocatalytic activity than the pristine EV nanoparticles. EV nanoparticles were also loaded onto fluorine-doped graphene sheets to synthesize EV/FG24 nanocomposite; 2,4-dinitrophenol and phenol were completely mineralized in 10 h [22].

So far, only a few works, as mentioned above, have involved the construction of EV-based heterojunctions to improve its visible light photocatalytic activity. However, EV and its heterojunctions still exhibit relatively low photodegradation efficiency. Much more efforts should be made to ameliorate the photodegradation activity of EV. Therefore, we constructed EVC heterojunctions by loading EV nanoparticles onto ultrathin CN nanosheets. EVCs presented ultrathin and porous structures, which increased the specific surface area and reaction active sites, shortened the diffusion distance of photoinduced charge carriers. The formation of EVCs extended the visible light absorption of EV and accelerated the separation of charge carriers and ultimately enhanced the adsorption capability and photocatalytic removal efficiency of methylene blue (MB). The effects of ionic strength, initial concentration, temperature, and pH on the adsorption of MB were investigated. The adsorption isotherm and adsorption kinetics were fitted with different models. EVCs exhibited a synergy of adsorption and photocatalysis to remove MB.

## 2. Results and Discussion

### 2.1. Characterization of EVCs

**Figure 1:**
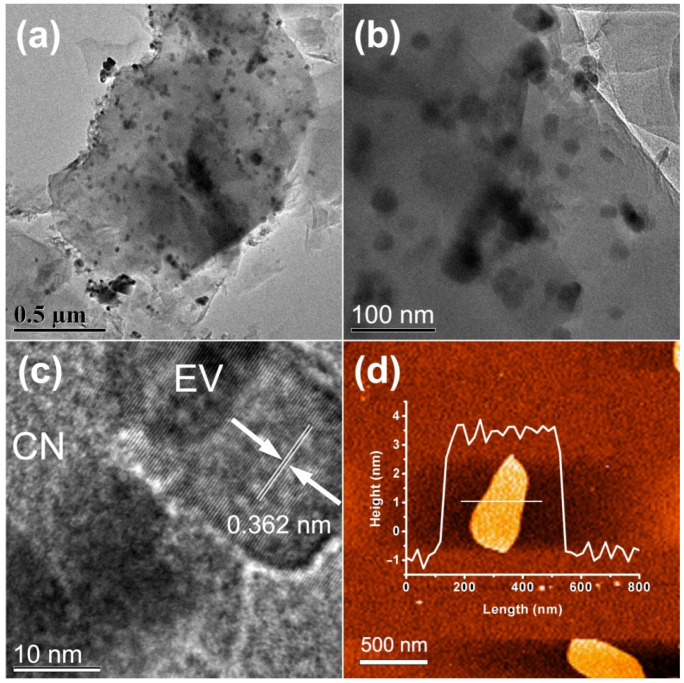
(**a**,**b**) TEM, (**c**) HRTEM, and (**d**) AFM images of EVC-2.

The morphology and textural properties of EVC-2 were determined by TEM and HRTEM. As displayed in Figure 1a,b, the ultrathin, layered nanosheet structure of CN was observed. The ultrathin layered structure significantly increased the specific surface area of EVC-2, which was further confirmed by BET measurements. It should be advantageous to boost the absorption capability of EVC-2 for MB. EV nanoparticles can be clearly observed in Figure 1a,b. The diameters of EV nanoparticles were 20–50 nm. EV nanoparticles were located on the surface of the CN nanosheets. Distinct lattice fringes can be observed in the HRTEM image (Figure 1c). The spacing of the lattice fringe was 0.362 nm, corresponding to the (200) crystal plane of tetragonal EV nanoparticle (PDF#15-0809) [14,17,18]. These results indicate that the intimate contact between EV nanoparticles and CN nanosheets successfully formed EVC heterojunctions [23]. Figure 1d shows the AFM image of EVC-2. The thickness of the CN nanosheets was approximately 4–5 nm, suggesting that EVCs had a higher specific surface area and more abundant adsorption sites than bulk g-C_3_N_4_. This was instrumental in promoting the adsorption capability. Moreover, the diffusion distance of the photoinduced charge carriers was shortened in the CN ultrathin nanosheets. It could reduce the recombination of photoinduced charge carriers and boost the photocatalytic performance [8].

**Figure 2:**
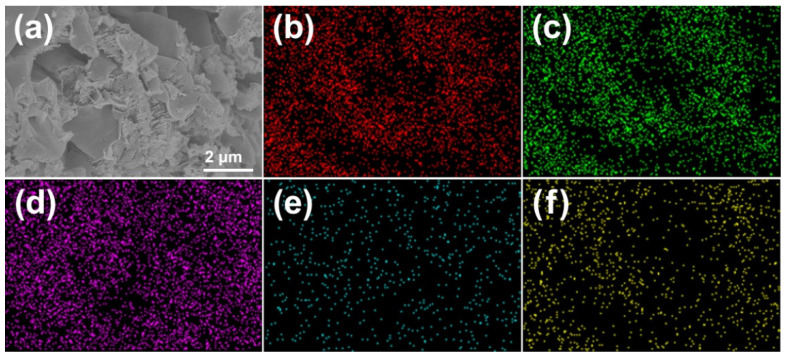
(**a**) SEMand element mapping images of (**b**) C, (**c**) N, (**d**) Eu, (**e**) V, and (**f**) O of EVC-2.

The morphology and chemical composition of EVC-2 were analyzed by SEM, EDS, and element mapping. From the SEM image displayed in Figure 2a, a stacked lamellar morphology emerged. It was the layered structural features of CN. The stacked CN of EVC-2 acquired a three-dimensional porous architecture. Therefore, EVC-2 manifested a mesoporous structure and large pore size distribution, endowing EVC-2 with an exceptional adsorption capability for MB. This has been confirmed by BET measurements. C, N, Eu, V, and O were detected by the energy dispersion spectrum in the selected region (Figure S1). The weight ratios of C, N, Eu, V, and O of EVC-2 are shown in Table S1. The mass percentage of EV in EVC-2 was calculated according to the EDS quantitative results. It was approximately 2.67%, which was slightly higher than the value estimated from the initial Eu(NO_3_)_3_ and NH_4_VO_3_ concentration in the precursor. This might be caused by the mass loss of CN in the reheating process. The uniformity of C, N, Eu, V, and O in EVC-2 was corroborated by elemental mapping, and all appeared in the elemental mapping images (Figure 2b–f). The uniformity of Eu, V, and O demonstrated that the EV nanoparticles were distributed evenly in EVC-2.

**Figure 3:**
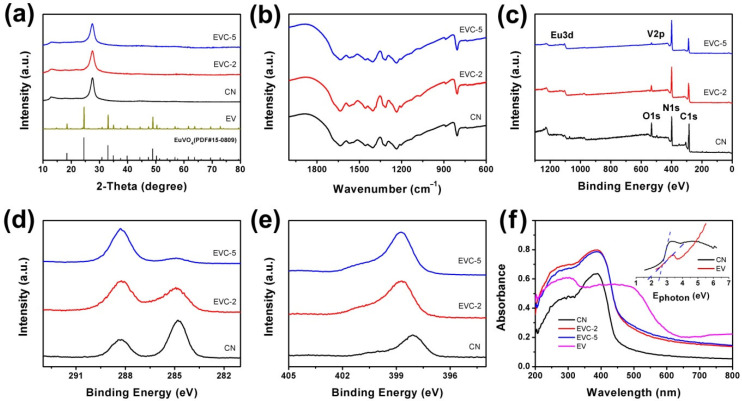
(**a**) XRD patterns, (**b**) FTIR spectra, (**c**) XPS survey spectra, high-resolution XPS spectra of (**d**) C1s, (**e**) N1s, and (**f**) UV-vis diffuse reflectance spectrum (Inset: band gap energy of CN and EV) of CN, EVC-2, and EVC-5.

The XRD patterns of CN, EVC-2, and EVC-5 are shown in Figure 3a. Two diffraction peaks at 12.8° and 27.7° were observed, corresponding to the (110) and (022) planes of CN (JCPDS#87-1526) [24]. The XRD diffraction peaks of EV nanoparticles were hardly detected in EVC-2 and EVC-5. This could be attributed to the limited dosage of EV in the heterojunctions. The small amount of EF could not be enough to change the chemical skeleton and bulk structure of CN, which is consistent with the results reported in the literature [25,26]. An adsorption band appearing between 2000 and 600 cm^−1^ was displayed in the FTIR spectra of CN, EVC-2, and EVC-5 (Figure 3b). The strong adsorption band located in the range of 1200 to 1700 cm^−1^ was generally originated from the C-N stretching vibrations of CN heterocycles [27]. The sharp band emerging around 805 cm^−1^ can be allocated to the breathing vibration of the s-triazine units of CN. After the adsorption of MB, the variation of the FTIR spectra of EVC-2 was almost undetectable (Figure S2), indicating its excellent structural stability [28]. In addition, the peak at 888 cm^−1^ is characteristic of the =C-H out-of-plane deformation vibration of MB [29].

Figure 3c depicts the XPS survey spectra of CN, EVC-2, and EVC-5. It reveals the existence of C, N, Eu, V, and O in EVC-2 and EVC-5. The O1s peak intensity of CN reduced with the increase of EV dosage, as reported previously, indicating more H_2_O being absorbed on CN [25,27]. In the C1s high-resolution XPS spectra of CN, EVC-2, and EVC-5, two peaks at 284.8 and 288.3 eV for CN were observed. The peaks at 284.8 eV originated from the adventitious carbon, and the peaks at 288.3 eV were derived from the sp^2^-bonded C in CN [27]. The N1s peak of CN at 398.1 eV shifted to 398.8 eV for EVC-2 and EVC-5. These peaks could be attributedto the sp^2^ nitrogen atoms in C–N=C. The increased N1s binding energy of EVC-2 and EVC-5 demonstrate the reduced electron cloud density of N atoms [30]. This indicates that the coordination of Eu or V ions with pyridinic N occurred where N provided lone-pair electrons and Eu^3+^ or V^5+^ supplied the unoccupied d orbit [31]. The XPS results also suggested that EVCs were successfully formed through the coordination interaction between Eu^3+^ or V^5+^ and N [32]. The construction of EVCs was advantageous to the transfer of photoinduced charge in the photocatalytic reaction, and thus improved the photocatalytic degradation efficiency of MB over EVCs. After the adsorption of MB, the C1s peaks of EVC-2 at 284.8 eV and 288.3 eV shifted to 284.5 eV and 287.6 eV, respectively. The N1s peak of EVC-2 at 398.8 eV shifted to 398.1 eV (Figure S3). This reveals the variation of the electron cloud density of C and N atoms after the adsorption, which could be attributed to the π–π electron donor-acceptor interaction between the CN nanosheets and the MBmolecules [33]. The absorption edge of CN and EV was 457 and 652 nm, respectively, corresponding to 2.71 and 1.90 eV of the band gap (Figure 3f). The UV-vis DRS revealed that EVC-2 and EVC-5 presented stronger visible light absorption than CN, which was instrumental in improving the utilization of visible light in the photocatalytic process. The absorption edge of EVC-2 and EVC-5 lay between the values of CN and EV, further confirming the successful formation of EVC heterojunctions.

**Figure 4:**
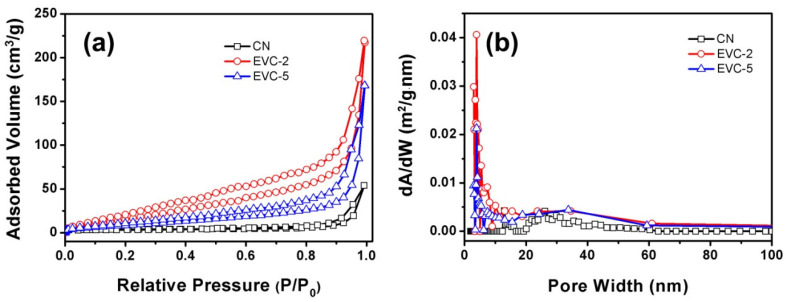
(**a**) The N_2_ adsorption–desorption isotherms and (**b**) the corresponding BJH pore size distribution curves of CN, EVC-2, and EVC-5.

The BET specific surface areas (S_BET_) and mesoporous structures of CN, EVC-2, EVC-5, EVC-10, and EVC-20 were assessed by the N_2_ adsorption–desorption isotherms and the corresponding BJH pore size distribution curves. All catalysts presented type IV isotherms (Figure 4a), which indicated the mesoporous structures of these catalysts [6,25,34]. The H3 hysteresis loop at high P/P_0_ from 0.50 to 1.00 suggested that the mesopores of the samples were irregular. The S_BET_ of EVC-2 (80.43 m^2^ g^−1^), EVC-5 (38.14 m^2^ g^−1^), EVC-10 (36.21 m^2^ g^−1^), and EVC-20 (32.53 m^2^ g^−1^) were distinctly higher than that of CN (12.32 m^2^ g^−1^). The pore volumes of EVC-2 and CN were 0.15 and 0.02 cm^3^ g^−1^ (Table S2), respectively. These results reveal that the EVCs possessed higher specific surface areas and more mesopores for the adsorption of contaminants. Meanwhile, this would imply that EVCs had more reaction-active sites than CN for the photocatalytic degradation of MB. The BJH pore size distribution curves of CN, EVC-2, and EVC-5 are depicted in Figure 4b, indicating the remarkable increase of EVC-2 and EVC-5 in the range of 1–50 nm. This might be caused by thermal etching in the reheating process, which is similar to the previous reported results [25]. The ultrathin layered structures increased the specific surface area of EVC-2, which is confirmed by the TEM and AFM results in Figure 1. The stacked CN of EVC-2 acquired a three-dimensional porous architecture, which is corroborated by the SEM results in Figure 2. This further indicates that EVC-2 and EVC-5 possess a higher pore size distribution ranging from 1 to 50 nm. This increased mesoporous structure endows EVC-2 with superior adsorption capability for MB in wastewater [23].

### 2.2. Adsorption Kinetics and Isotherm of MB

**Figure 5:**
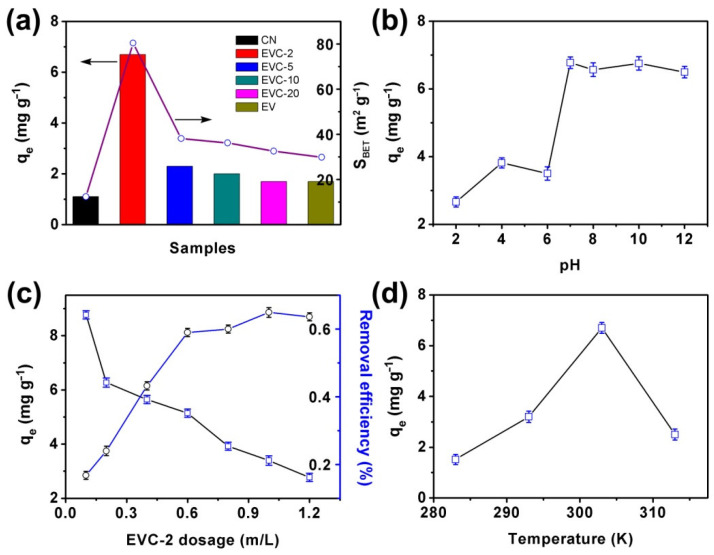
(**a**) Adsorption of MB on CN, EV, and EVCs: the impact of (**b**) pH, (**c**) initial dosage, and (**d**) temperature on the MB adsorption over EVC-2.

CN, EV, and EVCs were used to adsorb MB. The effect of different EV mass ratios on the MB adsorption capacity was investigated (Figure 5a). EV content in EVCs significantly affected the adsorption amount of MB. The q_e_ values of EVCs were higher than that of CN (1.1 mg g^−1^). EVC-2 exhibited a highest MB adsorption capacity of 6.75 mg g^−1^, which was over six times that of CN. This result was well consistent with the BET measurements, as displayed in Figure 5a. It suggested that the reheating synthesis approach was an effective way to ameliorate the adsorption capacity of EVCs. The thermal etching approach could be instrumental in obtaining an ultrathin, layered structure of EVCs and increasing its specific surface area. The adsorption capacity and specific surface areas decreased with the increase of EV content. This might originate from more adsorption active sites being occupied by EV nanoparticles.

MB adsorption capacity with different solutions of pH over EVC-2 at 30 °C was tested and is depicted in Figure 5b. It revealed that the adsorption capacity increased remarkably while the pH was higher than 6. At a pH of less than 6, the adsorption capacity was extremely low. It reached 6.75 mg g^−1^ when pH was 7. The alkaline condition was advantageous to the MB adsorption, which is similar to many reported results [35,36,37]. These results indicated that the surface charge of EVC-2 was remarkably affected by the solution pH. Electrostatic interaction between EVC-2 and MB contributed to adsorption capacity. MB is a cationic dye and is easily adsorbed on a negatively-charged adsorbent [35]. The point of zero charge pH (pH_pzc_) of EVC-2 was 6.48 (Figure S4). This suggests that the EVC-2 was negatively charged at pH > 6.48 and positively charged at pH < 6.48. The negatively-charged surface of EVC-2 at a higher pH would enhance the electrostatic interaction between EVC-2 and MB, which remarkably increased the adsorption capacity. The electrostatic repulsion between positively-charged EVC-2 and MB resulted in a low adsorption capacity of MB at a solution pH of less than 6.

The effect of initial EVC-2 dosage on MB adsorption at 30 °C was studied and is presented in Figure 5c. The experiment was conducted after 72 h of equilibrium under continuous shaking in the dark. The initial MB concentration was 5 mg L^−1^. It can be seen from Figure 5c that the removal efficiency of MB increased when the dosage of EVC-2 was raised, which is related to the increased active sites of the adsorbent [38]. As the EVC-2 dosage increased from 0.1 g L^−1^ to 1.2 g L^−1^, the adsorption capacity of MB was extremely abated from 8.77 mg g^−1^ to 2.77 mg g^−1^. The reason for this result is mainly that the initial MB concentration remained unchanged in the solution. With the increase of EVC-2 dosage, the adsorption active sites on EVC-2 were redundant and could not be fully utilized [39]. The effect of temperature on MB adsorption capacity at 10, 20, 30, and 40 °C is illustrated in Figure 5d. The adsorption capacity was enhanced when the temperature rose from 10 to 30 °C, implying it was an endothermic process resulting from the π–π interaction between EVC-2 and MB [25]. MB had relative weak molecular thermal motion at low temperature, resulting in fewer molecules being diffused to the EVC-2 surface. The adsorption capacity was consequently less at lower temperatures. With the increase of temperature, MB absorbed the heat and accelerated molecular thermal motion, thus exhibitinga higher adsorption capacity [40,41]. The adsorption capacity decreased at 40 °C. This might be caused by the desorption effect due to the higher velocity of the molecular thermal motion at 40 °C.

**Figure 6:**
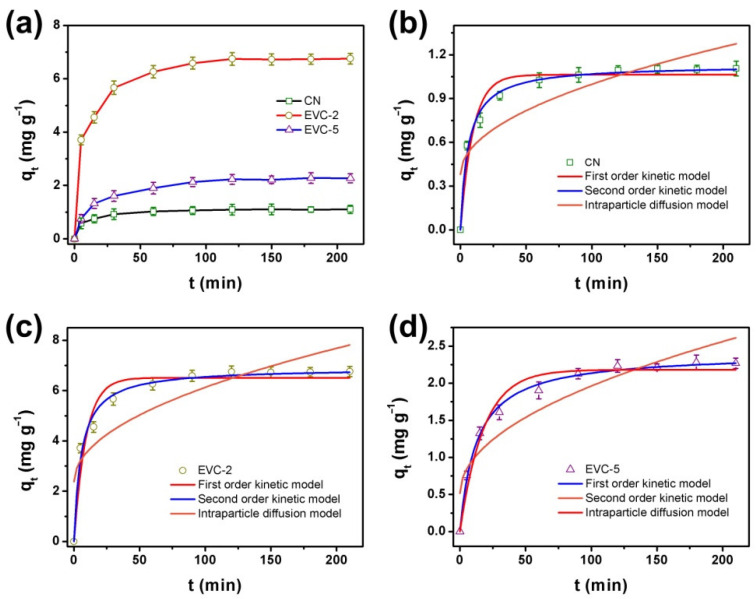
(**a**) The adsorption kinetic curves of MB (5 mg L^−1^) on CN, EVC-2, and EVC-5: the first order, second-order, and intraparticle diffusion kinetics model for adsorption of MB on (**b**) CN, (**c**) EVC-2, and (**d**) EVC-5.

The adsorption kinetics curves of MB on CN, EVC-2, and EVC-5 are displayed in Figure 6a. The adsorption equilibrium of MB on CN, EVC-2, and EVC-5 was reached in 60 min. The adsorbed amount of MB was 1.09, 6.75, and 2.27 mg g^−1^, respectively. The adsorption capability was enhanced after the formation of EVCs. The pseudo first-order, pseudo second-order, and intraparticle diffusion kinetics model were adopted to fit the experimental data for the elaboration of the kinetics mechanism of the adsorption process [42]. As can be clearly seen in Figure 6b–d, the adsorption data well met the pseudo second-order kinetics model. The corresponding R^2^ and RMSE values are listed in Table S3. Therefore, the MB adsorption on CN, EVC-2, and EVC-5 all followed the pseudo second-order kinetics model. This demonstrates that the adsorption rate of MB on the as-prepared adsorbents was controlled by the chemical adsorption, which was the rate-determining step of the adsorption process [43]. The electron transfer or electron sharing between the adsorbent and MB was the primary driving force of the adsorption.

The MB adsorption kinetic parameters from the fitted pseudo second-order equation are displayed in Table S4. The calculated adsorption capacity values were consistent with the experimental values obtained in Figure 6a. EVC-2 possessed the highest adsorption capacity, which was 6.2 times that of CN. This reveals that the formation of EVCs could significantly enhance the adsorption capability. The enhancement of the adsorption capability of EVC-2 and EVC-5 might originate from thermal etching in the reheating process, which would increase the specific surface areas through reducing the thickness of the CN nanosheets and the formation of loose-stacked porous architecture. The SEM and BET results confirmed the significant increase of the pore of EVCs in the whole range of 1–50 nm. The increased specific surface areas could generate more surface reaction sites. As a result, the adsorption and photocatalytic degradation performance would be extremely enhanced.

**Figure 7:**
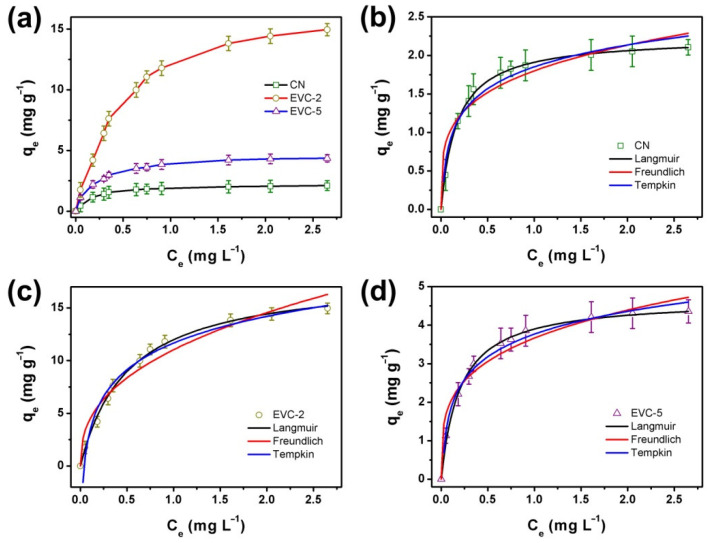
(**a**) Adsorption isotherms of MB (5 mg L^−1^) on CN, EVC-2, and EVC-5: Langmuir, Freundlich, and Tempkin isotherm model for the adsorption of MB on (**b**) CN, (**c**) EVC-2, and (**d**) EVC-5.

An adsorption isotherm can be employed to provide information regarding the equilibrium adsorptions. The adsorption capacity increased with the equilibrium concentration of MB (Figure 7a). The experimental equilibrium adsorption data of MB on CN, EVC-2, and EVC-5 were measured and fitted by the Freundlich, Temkin, and Langmuir isotherm models [25,35]. The corresponding R^2^ and RMSE values are listed in Table S5. As presented in Figure 7b–d, the equilibrium adsorption data conformed to the Langmuir isotherm model according to the correlation coefficients (R^2^ and RMSE) revealed in Table S5. This result indicates that the adsorption of MB on the surface of CN, EVC-2, and EVC-5 was monolayer adsorption [2]. The adsorption isotherm parameters of MB on CN, EVC-2, and EVC-5 are presented in Table S6. The q_m_ calculated from the Langmuir isotherm model were 2.24, 20.0, and 4.76 mg g^−1^ for CN, EVC-2, and EVC-5, respectively. This is very similar to the experimental equilibrium adsorption values obtained from Figure 7a. The Langmuir constant (KL) was also calculated and is listed in Table S6; it suggests that EVC-2 had a highest affinity for the adsorption of MB on its surface binding sites.

### 2.3. Adsorption and Photocatalytic Degradation of MB

**Figure 8:**
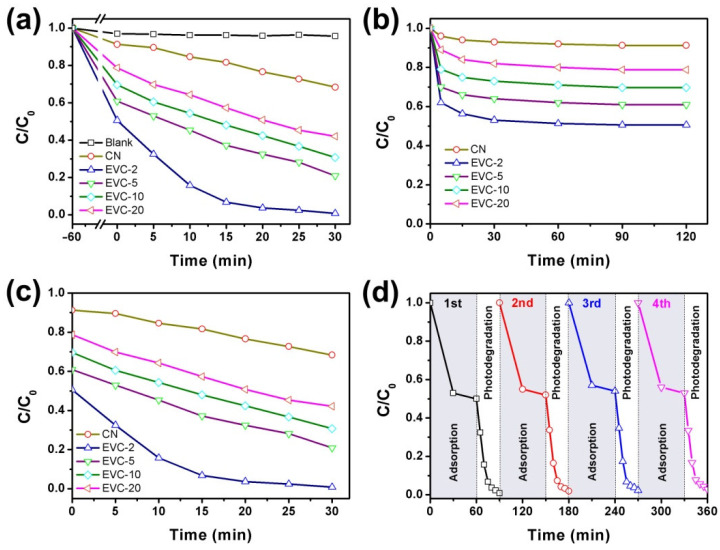
(**a**) The removal of MB over the as-prepared catalysts under visible light irradiation, (**b**) the adsorption of MB over the catalysts in the dark, (**c**) the photodegradation of MB over the catalysts under visible light irradiation, (**d**) the synergistic removal of MB over EVC-2 in the recycle experiment.

The adsorption–photocatalysis synergistic process of MB on the as-prepared catalysts under visible light irradiation was investigated and is illustrated in Figure 8a. As revealed in this figure, the formation of EVCs remarkably improved the removal efficiency of MB. The optimal removal efficiency of MB over EVC-2 ultimately reached 100% after 30 min of degradation. It was 22.7 times higher than that of mere photolysis of MB. All EVC samples presented better MB removal performance than CN, suggesting the higher separation efficiency of photoinduced charge carriers in EVCs. The removal of MB in this degradation process could be considered as the synergistic effect of adsorption and photocatalysis. There was approximately 22–50% of MB eliminated by the adsorption process before light irradiation over EVCs. By comparison, only 8.7% of MB was removed over CN in 60 min of the adsorption process. This confirms that EVCs possess much stronger MB adsorption ability than CN. The same results were also observed in the previous work [25] and are clarified in Figure 6 and Figure 7. As shown in Figure 8b, the adsorption–desorption equilibrium was obtained within 60 min. The removal efficiency of MB reached 49.5% after 60 min of the adsorption process on EVC-2 (Figure 8b), which was 5.7 times that of CN. The photocatalysis process over the as-prepared catalysts was investigated and is exhibited in Figure 8c. Before photocatalytic degradation, the adsorption–desorption equilibrium was achieved first, and thus the influence of the adsorption on the photocatalytic process was ignored. There was approximately 50.5% of MB removed in the photocatalytic degradation process on EVC-2. This was 2.2 times that of CN. These results demonstrate that EVC-2 had optimum adsorption and photocatalytic performance.

The practical application of the catalysts for the removal of contaminants requires the as-prepared sample presented to have stable degradation properties. Therefore, the stability of EVC-2 was implemented by four cycles of the adsorption and photocatalysis experiments. EVC-2 was separated by centrifugation, desorbed in deionized water, and dried at 60 °C after each cyclic experiment. The stability experimental results are displayed in Figure 8d. Only 5% of adsorption and 3% of photocatalysis capability was lost after four cycles of degradation experiments. TEM, FTIR, and XRD (Figures S5 and S6) were measured to investigate the variations of the morphology and the crystal structure between the fresh and used EVC-2. No obvious changes were found between the fresh and used EVC-2. This proves that EVC-2 was highly stable for practical utilization. In addition, TOC analysis was employed to clarify the mineralization efficiency of MB on EVC-2 under visible light irradiation. As shown in Figure S7, the TOC decreased to 42.4% after 30 min of the adsorption and photocatalysis process, corresponding to a mineralization efficiency of 57.6%. In contrast, with 100% photocatalytic degradation efficiency, the lower mineralization efficiency indicated that the mineralization of MB was slower than the decolorization process. It was the degradation intermediates that caused the relatively higher TOC value [44,45]. The mineralization of MB to completely generate CO_2_ and H_2_O needed to react for much longer than 2 h under visible light illumination.

### 2.4. Photocatalytic Kinetics of MB

**Figure 9:**
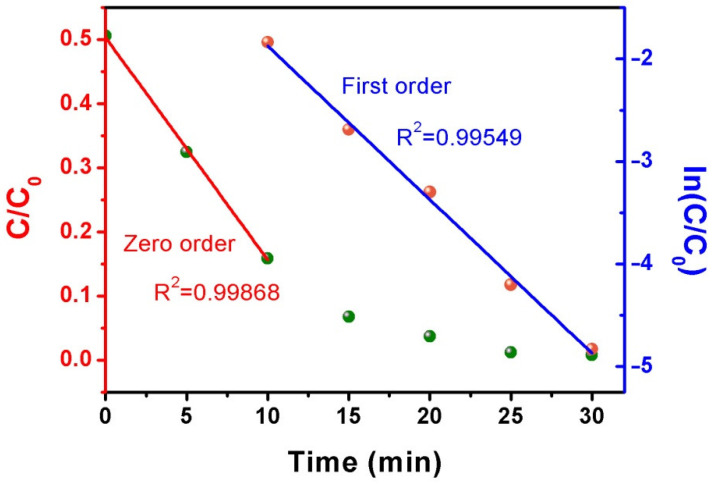
The photocatalytic degradation of MB on EVC-2 under 40 W LED irradiation: the corresponding zero-order kinetics curve (left) and the first-order kinetics curve (right).

The photocatalytic degradation of MB on EVC-2 under visible light irradiation is depicted in Figure 9. It obviously manifested two types of kinetics characteristics in the different degradation times. The photocatalytic degradation data obeyed the zero-order kinetics equation in the range of 0–10 min (Figure 9, left). In this stage, the photocatalytic degradation reaction rate was almost extraneous to MB concentration. This might originate from the excessive MB in the initial degradation solution. In the meantime, the photocatalytic degradation reaction rate only related to the amount of surface active sites of the photocatalyst [24]. The zero-order degradation reaction rate constant (k_0_) was 0.035 mg L^−1^ min^−1^. With the gradual decrease of MB concentration after 10 min of degradation, the MB diffusion rate from the solution to the surface of the photocatalyst was reduced. The influence of the MB concentration could not be disregarded at this stage. The photocatalytic degradation data consequently followed the first-order kinetics equation for 10–30 min (Figure 9, right). The first-order degradation reaction rate constant (k_1_) was 0.150 min^−1^. As shown in Figure 8a, the adsorption and photocatalytic performances of MB on CN, EVC-5, EVC-10, and EVC-20 were relatively low. All photocatalytic degradation data seemed to comply with the zero-order kinetics equation during the whole degradation period (Figure 8c). This might be caused by the comparatively low photocatalytic degradation reaction rate of MB on these samples, resulting in the excessive MB concentration over 30 min. The k_0_ values of MB on CN, EVC-5, EVC-10, and EVC-20 were 0.007, 0.013, 0.013, and 0.012 mg L^−1^ min^−1^, respectively (Figure S8). The k_0_ of EVC-2 was five times that of CN.

### 2.5. Active Oxidation Species and Possible Mechanism

**Figure 10:**
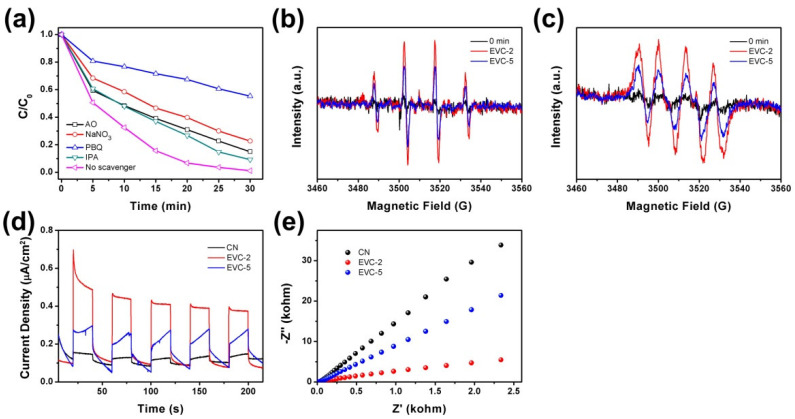
(**a**) the effect of radical scavengers on MB photocatalytic degradation on EVC-2 under 40 W LED irradiation: the ESR spectra of (**b**) DMPO-•OH in water; (**c**) DMPO-•O_2_^−^ in methanol with EVC-2 and EVC-5 after 15 min of visible light irradiation; (**d**) TPC spectra; (**e**) EIS Nyquist plots of CN, EVC-2, and EVC-5.

Radical scavenger experiments were conducted to identify the major reactive species in the MB photocatalytic degradation on EVC-2 (Figure 10a). NaNO_3_, AO, IPA, and PBQ were adopted as the scavengers to trap e^−^, h^+^, •OH, and •O_2_^−^, respectively [46]. The degradation efficiencies of MB on EVC-2 were obviously decreased with the presence of NaNO_3_, AO, IPA, and PBQ, indicating that e^−^, h^+^, •OH, and •O_2_^−^ were all involved in the photocatalytic degradation process. In particular, when PBQ was added to the degradation solution, the degradation efficiency was enormously reduced, indicating that •O_2_^−^ was absolutely the major active oxidation species in MB degradation. As can be seen in the radical scavenger experimental results shown in Figure 10a, the influence of the reactive species followed the order of •O_2_^−^> e^−^> h^+^> •OH, and the degradation efficiencies of MB on EVC-2 were decreased to 45%, 77%, 85%, and 91%, respectively. The degradation efficiency declined slightly in the presence of NaNO_3_, AO, and IPA, suggesting that e^−^, h^+^, and •OH played only a relatively minor role in the MB degradation process. The ESR spectra of DMPO-•OH and DMPO-•O_2_^−^ were employed to further confirm the generation of active species •OH and •O_2_^−^ radicals in the MB photocatalytic degradation process on EVC-2 and EVC-5 (Figure 10b,c). The ESR signals of DMPO-•OH and DMPO-•O_2_^−^ adducts could be clearly detected after 15 min of visible light irradiation, but were not detectable in the dark. This signifies that the •OH and •O_2_^−^ radicals were only produced in the photocatalytic degradation process. In addition, The DMPO-•OH and DMPO-•O_2_^−^ signal intensities of EVC-2 were much stronger than that of EVC-5, revealing that EVC-2 had higher •O_2_^−^ generation capability than EVC-5. This is consistent with the photodegradation results presented in Figure 8c, which indicate that EVC-2 possessed higher carrier separation efficiency. Based on the above-mentioned experimental results, the possible removal mechanism of MB on EVC-2 can be deduced. MB molecules were first adsorbed on the surface of EVC-2. There followed the visible light absorption and the photoinduced electron and hole generation. Then, the photoinduced electron and hole separated and transferred to the surface of EVC-2 and produced •OH and •O_2_^−^. Next, MB was ultimately degraded into small molecules by the complex reaction with •O_2_^−^, e^−^, h^+^, and •OH. The formation of ultrathin nanosheet structures and EVC heterojunctions could provide the driving force for the separation and transfer of photoinduced electron-hole pairs.

Transient photocurrent response (TPC) and electrochemical impedance spectroscopy (EIS) was further conducted to confirm the photoinduced charge separation efficiency of CN, EVC-2, and EVC-5. As shown in Figure 10d, EVC-2 and EVC-5 had higher photocurrent density than CN, indicating that the formation of EVCs promoted charge separation and transfer efficiency. EIS Nyquist plots of EVC-2 and EVC-5 possessed a smaller arc radius than CN, confirming the same conclusion as the TPC result (Figure 10e). In addition, EVC-2 exhibited a higher photocurrent density and a smaller arc radius than EVC-5, clarifying that EVC-2 had higher charge separation efficiency and superior photocatalytic degradation efficiency than EVC-5. This is consistent with the photocatalytic degradation result of MB over as-prepared catalysts.

In this study, the superior photocatalytic degradation activity of EVC-2 was primarily attributed to the formation of an ultrathin EVCs heterojunction structure and the synergistic effect of adsorption and photocatalysis. We firstly manufactured CN nanosheets with an ultrathin structure by thermal etching and ultrasonic techniques. This thus increased its specific surface area and reaction active sites and shortened the diffusion distance of photoinduced charge carriers. Further, EV nanoparticles were loaded onto CN nanosheets to form an EVCs heterojunction. This consequently accelerated the separation of charge carriers and reduced its recombination. These factors boosted the generation of e^−^, h^+^, •OH, and •O_2_^−^, which remarkably ameliorated the visible light-driven photocatalytic degradation efficiency. Based on these advantages, EVC-2 emerged with a much higher adsorption capacity and photocatalytic activity than that of pristine CN.

## 3. Materials and Methods

### 3.1. Synthesis of EVCs

Bulk g-C_3_N_4_ was synthesized by the polycondensation of dicyandiamine and NH_4_Cl, with a 1:1 mass ratio at 550 °C for 4 h according to the previous literature, with certain modifications [47]. The light-yellow powder was collected and ground. An amount of 100 mg of g-C_3_N_4_ was mixed with the 100 mL deionized water and underwent ultrasonic treatment for 4 h [48]. The mixture was centrifuged at 4000 rpm for 20 min. The g-C_3_N_4_ nanosheets in the supernatant were separated by vacuum freeze-drying.

An amount of 200 mg of g-C_3_N_4_ nanosheets and a certain amount of Eu(NO_3_), and NH_4_VO_3_ were mixed in 100 mL of deionized water for 2 h. The water was then evaporated and the obtained solid product was ground. EVCs powder was produced after heating the powder for 2 h at 550 °C. The as-prepared samples were named as EVC-2, EVC-5, EVC-10, and EVC-20, respectively, while 2, 5, 10, and 20 wt% of EV was contained in the EVCs. EV was generated under the same reaction conditions except for the absence of g-C_3_N_4_ nanosheets.

### 3.2. Catalyst Characterization

Transmission electron microscopy (TEM, JEOL, Tokyo, Japan) and high-resolution TEM (HRTEM) images were taken on a JEOL-2100F transmission electron microscope. Scanning electron microscopy (SEM, JEOL, Tokyo, Japan) images, energy dispersion spectrum (EDS), and elemental mapping images of the samples were taken on a JSM-4800F scanning electron microscope. Atomic force microscopy (AFM, Bruker, Billerica, MA, USA) images were measured on a Bruker Multimode 8 AFM system. X-ray diffraction (XRD, Rigaku Corporation, Tokyo, Japan) patterns of g-C_3_N_4_, EF, and as-prepared EFC heterojunctions were taken on a Rigaku Smartlab diffractometer. Fourier-transform infrared spectra (FTIR, ThermoFisher, Waltham Mass, MA, USA) were recorded on a Nicolet NEXUS 470 spectrometer in the range of 4000–400 cm^−1^. XPS spectra (ThermoFisher, Waltham, MA, USA) were detected on a Thermo ESCALAB 250XI X-ray photoelectron spectroscopy spectrometer equipped with anAlKαX-ray source. The N_2_ adsorption desorption isotherms and BET specific surface area was measured on a Micromeritics ASAP 2460 analyzer at 77 K (Micrometrics, Londonderry, NH, USA).

### 3.3. Adsorption of MB

The adsorption experiment of MB was carried in the dark. Briefly, a 50 mg catalyst was mixed with 100 mL of different concentrations of MB. The pH value of the MB solution was adjusted to 7 using 0.1 M HCl or NaOH. The mixture solution was agitated in the dark at 150 rpm. An amount of 5 mL adsorption solution was added at certain intervals and removed the catalyst through 0.22 μm PTFE filter membrane. The MB concentration was determined on a UV-vis spectrophotometer at the absorption wavelength of 664 nm. The adsorption capacity (q_t_) at a given adsorption time was calculated. The impact of pH on MB adsorption over the catalyst was studied from 2 to 11 using 0.1 M HCl or NaOH to adjust the pH value. The effect of temperature on MB adsorption over the catalyst was investigated at 20, 30, and 40 °C, respectively. The adsorption kinetic data were fitted by a pseudo first-order and pseudo second-order adsorption kinetics model and intraparticle diffusion (Weber–Morris) model. Langmuir, Freundlich, and Tempkin models were selected to fit the adsorption isotherm data.

### 3.4. Photocatalytic Degradation of MB

The visible light driven photocatalytic degradation of MB was conducted under the irradiation of a 40 W LED. Typically, a 100 mg catalyst was mixed with 200 mL 5 mg L^−1^ MB solution. The mixture was stirred in the dark for 1 h for the adsorption–desorption equilibrium of MB over the catalysts. An amount of 5 mL degradation suspension was taken out at certain intervals and removed the catalyst through 0.22 μm PTFE filter membrane. The MB concentration was determined on a UV-vis spectrophotometer by absorption at 664 nm. The photocatalytic degradation kinetics curve was obtained by plotting degradation efficiency against degradation time. The photocatalytic stability of the catalyst was performed by four cycles of degradation experiments. After each cycle, the catalyst was separated by centrifugation and washed thoroughly with ethanol and deionized water to eliminate the MB absorbed on the catalyst. The catalyst was then freeze-dried and collected for the next cycle of degradation experiments. To confirm the active species, NaNO_3_, AO (ammonium oxalate), IPA (isopropyl alcohol), and PBQ (p-benzoquinone) were adopted as the scavengers to trap e^−^, h^+^, •OH, and •O_2_^−^, respectively.

## 4. Conclusions

In summary, ultrathin CN nanosheets with a thickness of 4–5 nm were first manufactured by thermal etching and ultrasonic techniques. EV nanoparticles were then loaded onto the CN nanosheets to form EVCs. The as-prepared EVC-2 possessed the optimal adsorption and photocatalytic removal performance. The superior photocatalytic degradation activity of EVC-2 was primarily attributed to the formation of the ultrathin EVCs heterostructure and the synergistic effect of adsorption and photocatalysis. The MB adsorption capability of EVC-2 was 6.2 times that of CN, and the zero-order degradation reaction rate constant (k_0_) was 5 times that of CN. The MB adsorption on EVC-2 followed the pseudo second-order kinetics model, and the adsorption isotherm data complied with the Langmuir isotherm model. The photocatalytic degradation data of MB on EVC-2 obeyed the zero-order kinetics equation in the range of 0–10 min and abided by the first-order kinetics equation for 10–30 min. By comparison, the photocatalytic degradation data observed the zero-order kinetics equation during the whole degradation period. The radical scavenger experiments demonstrated that •O_2_^−^, e^−^, h^+^, and •OH were involved in the photocatalytic degradation process.

## Data Availability

The original data are available from the corresponding author upon reasonable request.

## Supplementary Materials

The supporting information can be downloaded at: https://www.mdpi.com/article/10.3390/ijms231710003/s1.
