# Supplementary material for: Effective Removal of Methylene Blue on EuVO4/g-C3N4 Mesoporous Nanosheets via Coupling Adsorption and Photocatalysis
Source: Int J Mol Sci. 2022 Sep 2;23(17):10003. doi: 10.3390/ijms231710003 (PMC9456035; doi:10.3390/ijms231710003)
Supplement: Supplementary file 1 [file ijms-23-10003-s001.zip › ijms-1875133-supplementary.pdf]

Effective removal of Methylene blue on $EuVO_4/g\text{-}C_3N_4$ mesoporous nanosheets via coupling adsorption and photocatalysis

Xia Ran, Li Wang, Bo Xiao, Li Lei, Jinming Zhu, Zuoji Liu, Xiaolan Xi, Guangwei Feng, Rong Li, Jian Feng*

Engineering Research Center for Molecular Medicine, School of Basic Medical Sciences, Guizhou Medical University, Guiyang 550025, China

* Corresponding author. Tel.: +86 851 88174017
E-mail address: jfeng@gmc.edu.cn (Jian Feng)

Table S1. EDS Quantitative results

Element	wt %	atm %
C	32.51	36.23
N	60.76	59.63
Eu	1.52	0.10
V	0.51	0.10
O	4.69	3.93

Table S2. BET surface area and pore size of CN, EV, EVC-2 and EVC-5

Sample	S_{BET} (m^2/g)	Pore volume (cm^3/g)	Pore diameter (nm)
CN	12.32	0.02	12.80
EV	49.89	0.07	5.65
EVC-2	80.43	0.15	7.44
EVC-5	38.14	0.08	8.83

Table S3. The R^2 and RMSE values obtained from fitting the adsorption curves of MB by different kinetic models

Adsorbent	Pseudo first order adsorption kinetics model		Pseudo second order adsorption kinetics model		Intraparticle diffusion model	
	R^2	RMSE	R^2	RMSE	R^2	RMSE
CN	0.95491	0.07532	0.99179	0.03214	0.71495	0.18937
EVC-2	0.94532	0.50591	0.98694	0.24725	0.70537	1.17431
EVC-5	0.95855	0.13680	0.99477	0.05576	0.84789	0.30086

Table S4. Adsorption kinetic parameters of MB on CN, EVC-2 and EVC-5

Adsorbent	pseudo second-order			q_e (exp) (mg g^{-1})
	q_e (cal) (mg g^{-1})	K_2 (g mg^{-1} min^{-1})	R^2	
CN	1.14	0.135	0.992	1.09
EVC-2	6.99	0.023	0.987	6.75
EVC-5	2.44	0.031	0.995	2.27

Table S5. The R^2 and RMSE values obtained from fitting the adsorption isotherm of MB by Tempkin, Freundlich and Langmuir isotherm model on CN, EVC-2 and EVC-5

Sample	Langmuir isotherm model		Freundlich isotherm model		Tempkin isotherm model	
	R^2	RMSE	R^2	RMSE	R^2	RMSE
CN	0.99824	0.02889	0.92703	0.18584	0.97141	0.11633
EVC-2	0.99730	0.26784	0.95722	1.06568	0.98118	0.70682
EVC-5	0.99633	0.08496	0.95934	0.28261	0.98964	0.14268

Table S6. Adsorption isotherms parameters of MB on CN, EVC-2 and EVC-5

adsorbent	Langmuir isotherm model		
	Q_m (mg g^{-1})	K_L (L mg^{-1})	R^2
CN	2.24	5.72	0.998
EVC-2	20.0	2.50	0.997
EVC-5	4.76	5.25	0.996

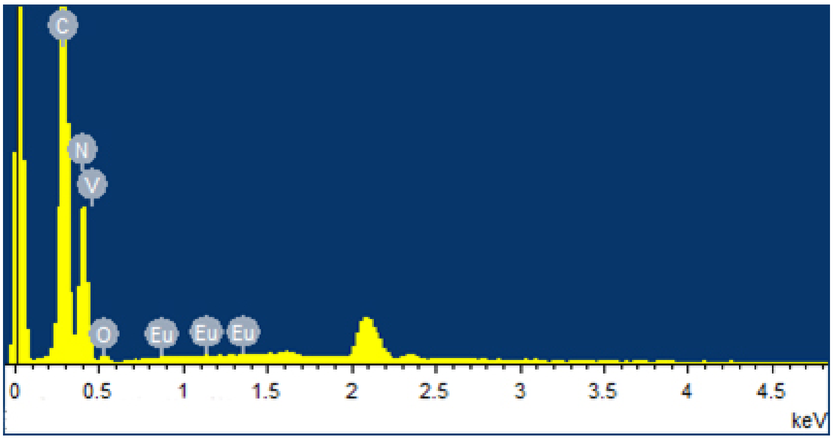

Figure S1 EDS spectrum of EVC-2

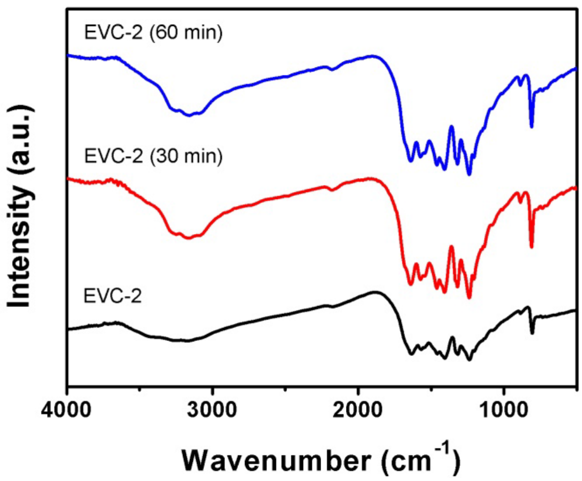

Figure S2 FTIR spectra of EVC-2 before and after the adsorption of MB with different time

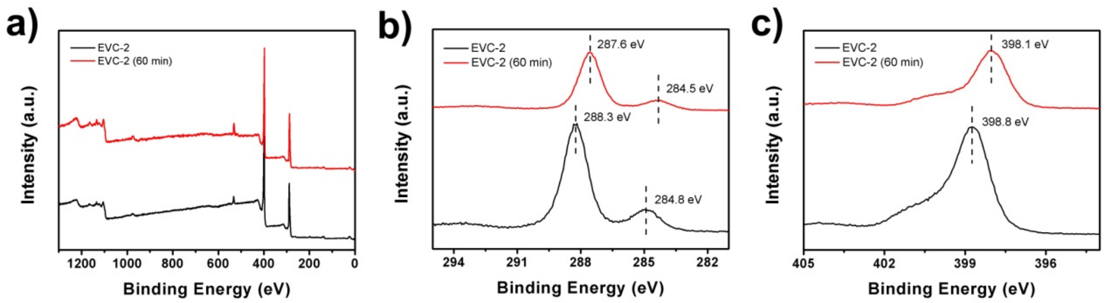

Figure S3 XPS spectra of EVC-2 before and after the adsorption of MB

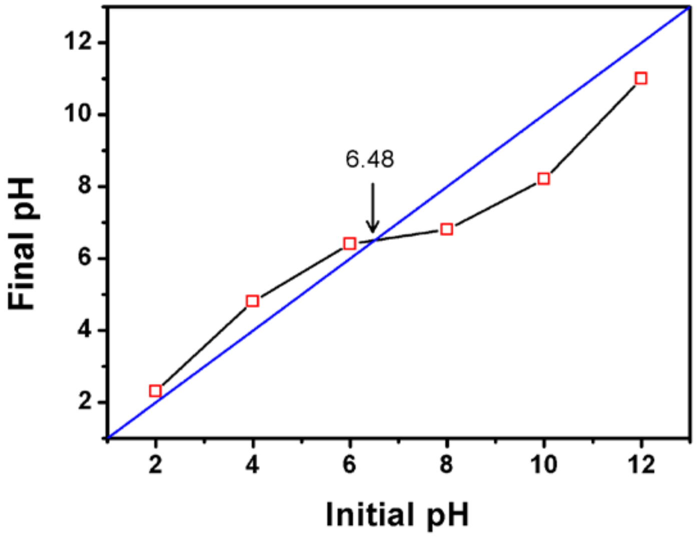

Figure S4 The point of zero charge (pH_{pzc}) of EVC-2: Bisector method

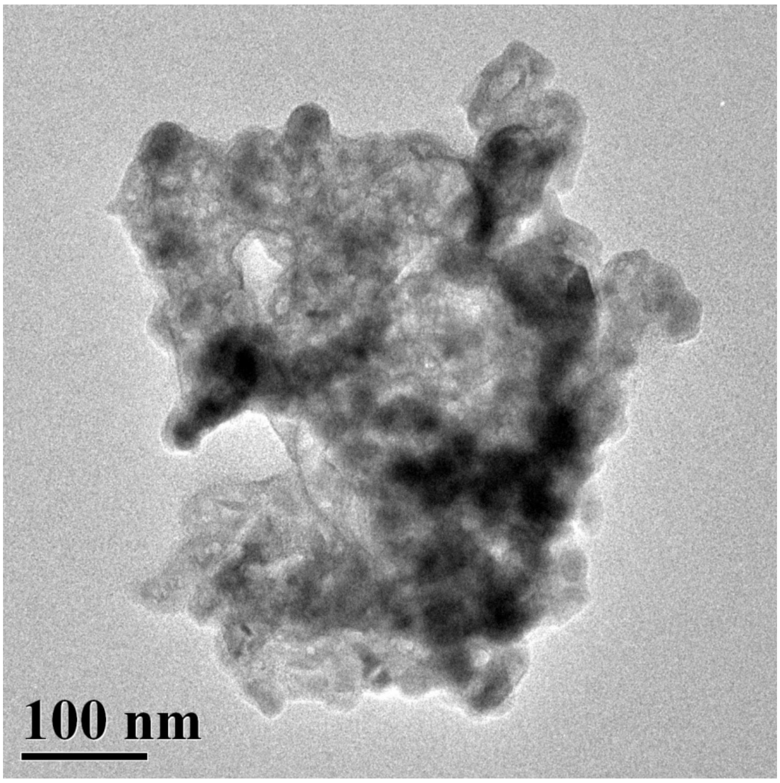

Figure S5 TEM image of used EVC-2

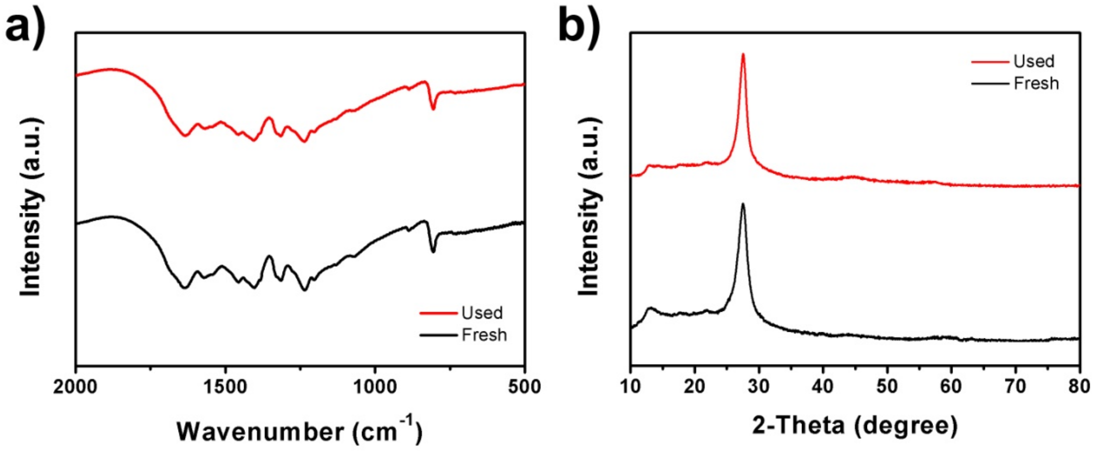

Figure S6 a) FTIR spectra and b) XRD patterns of fresh and used EVC-2

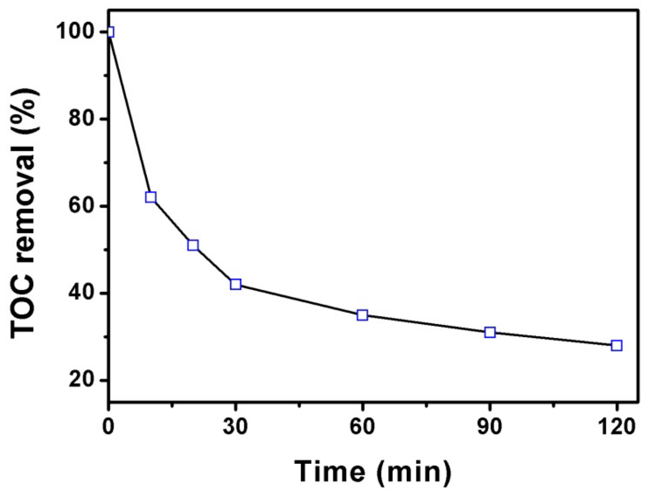

Figure S7 TOC removal efficiency of MB over EVC-2

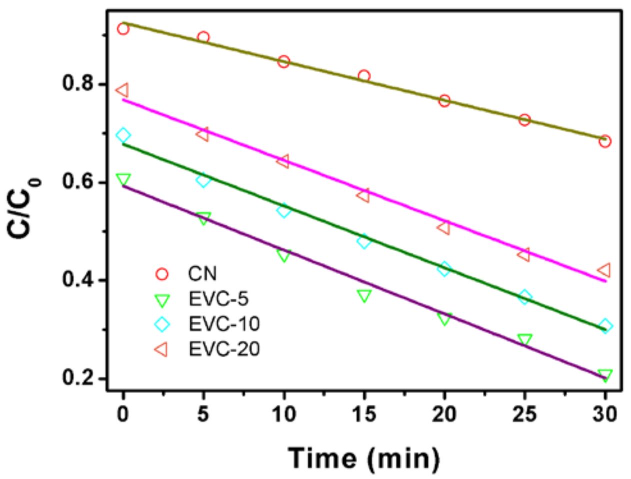

Figure S8 Zero order kinetics curve of MB degradationon on CN, EVC-5, EVC-10 and EVC-20
